# Digital Biomarkers for the Early Detection of Mild Cognitive Impairment: Artificial Intelligence Meets Virtual Reality

**DOI:** 10.3389/fnhum.2020.00245

**Published:** 2020-07-24

**Authors:** Silvia Cavedoni, Alice Chirico, Elisa Pedroli, Pietro Cipresso, Giuseppe Riva

**Affiliations:** ^1^Applied Technology for Neuro-Psychology Lab, Istituto Auxologico Italiano, Milan, Italy; ^2^Department of Psychology, Catholic University of the Sacred Heart, Milan, Italy; ^3^Faculty of Psychology, eCampus University, Novedrate, Italy

**Keywords:** gait analysis, kinematic, Mild Cognitive Impairment, Virtual Reality, Machine Learning, elderly, digital biomarkers, Artificial Intelligence

## Abstract

Elderly people affected by Mild Cognitive Impairment (MCI) usually report a perceived decline in cognitive functions that deeply impacts their quality of life. This subtle waning, although it cannot be diagnosable as dementia, is noted by caregivers on the basis of their relative’s behaviors. Crucially, if this condition is also not detected in time by clinicians, it can easily turn into dementia. Thus, early detection of MCI is strongly needed. Classical neuropsychological measures – underlying a categorical model of diagnosis - could be integrated with a dimensional assessment approach involving Virtual Reality (VR) and Artificial Intelligence (AI). VR can be used to create highly ecologically controlled simulations resembling the daily life contexts in which patients’ daily instrumental activities (IADL) usually take place. Clinicians can record patients’ kinematics, particularly gait, while performing IADL (Digital Biomarkers). Then, Artificial Intelligence employs Machine Learning (ML) to analyze them in combination with clinical and neuropsychological data. This integrated computational approach would enable the creation of a predictive model to identify specific patterns of cognitive and motor impairment in MCI. Therefore, this new dimensional cognitive-behavioral assessment would reveal elderly people’s neural alterations and impaired cognitive functions, typical of MCI and dementia, even in early stages for more time-sensitive interventions.

## Introduction

A categorical approach to diagnosing dementia struggles to capture subclinical conditions, such as Mild Cognitive Impairment (MCI). Crucially, MCI can either revert to normal cognition, stabilize, or slowly evolve toward other forms of dementia (Chiu, 2005; Walters, 2011; Morris, 2012; Díaz-Mardomingo et al., 2017; Vanacore et al., 2017). This construct indicates people affected by an in-between condition between normal aging and early dementia (Petersen, 2004; Albert et al., 2011; Mckhann et al., 2011; Seo et al., 2017) and is usually segmented into single- or multiple-domain amnestic (aMCI) and non-amnestic (naMCI) subtypes, depending on whether impairments concern only memory or other cognitive functions, e.g., executive and visuo-spatial abilities (Petersen, 2004; Apostolova and Cummings, 2008; Albert et al., 2011; Hughes et al., 2011; Michaud et al., 2017; Facal et al., 2019). Both patients and their caregivers can observe and report clear signals of this subtle waning, undiagnosable as dementia. Frequently, elderly people express concern over their perceived worsening in one or more cognitive domains, such as memory or language (Petersen et al., 1999, 2018). This waning has a great impact on their quality of life, reducing their ability to autonomously carry out activities. A key aspect concerns the possibility of detecting an initial cognitive decline at the behavioral level with a slowdown in execution of the instrumental activities of daily life (IADL), such as grocery shopping and medication and financial management (Kim and Kim, 2009; Gold and Gold, 2012).

Changes associated with subclinical forms of dementia manifest themselves through behavioral alterations. Usually, caregivers are the first ones to notice these altered behaviors, as shown by Van Vliet et al. (2011). The authors explored the barriers hindering a timely diagnosis of dementia, focusing on interviews conducted with caregivers of relatives that were later diagnosed with early-onset dementia (EOD). Caregivers frequently reported behavioral changes in relatives with EOD, either alone or associated with neuropsychiatric symptoms (NPS), such as apathy or depression, and personality changes. Behavioral impairment then evolved toward a decline in IADL and involved cognitive impairment, particularly memory loss (Van Vliet et al., 2011). The broader detrimental impact of behavioral changes generated familial/marital conflicts and reduced job productivity, leading to a decreased income or even dismissal (Van Vliet et al., 2011). Though valuable, this anecdotal information rarely becomes part of a (categorical) diagnosis based on medical and neuropsychological assessment. Over time, caregivers have been considered a source of information that is not always reliable, given their tendency to over- or under-estimate elderly people’s deficits, possibly due to knowledge gaps (Akl et al., 2015; Jekel et al., 2015). Caregivers might be absent or suffer from physical or psychological conditions exacerbated by their relative’s worsening (Okonkwo et al., 2008; Van Vliet et al., 2011; Pfeifer et al., 2013; Akl et al., 2015; Jekel et al., 2015). They might explain the elderly person’s decline and behavioral, cognitive, and personality changes rather as a result of aging. Sometimes, caregivers are not aware of the symptoms because of their relative’s ability to cover them up, denying their impairments or developing subsequent compensatory strategies to disguise the difficulties. This, in turn, delays the consultation of a practitioner and the diagnostic process as well (Okonkwo et al., 2008; Van Vliet et al., 2011; Roehr et al., 2019). Early detection of MCI, resulting in time-sensitive interventions, is still an open issue.

In this regard, two components appear relevant. Firstly, there is a need to rely more on rigorous and systematic behavioral analysis for early detection of MCI. Secondly, there is a need to integrate this new practice into current ones, i.e., neuropsychological evaluation. Including these data jointly in MCI assessment can allow a more sensitive measurement of the deficit by placing it on a continuum, reflecting a dimensional approach accounting for several other subclinical conditions, including Subjective Cognitive Decline (SCD; Roehr et al., 2019) or Pre-Mild Cognitive Impairment (Pre-MCI; Crocco et al., 2018; Grassi et al., 2018).

This is far more crucial when considering that MCI can turn into dementia if the elderly person does not receive a timely diagnosis (Chiu, 2005), which should be built upon finer discrimination among the early stages of MCI and the collection of behavioral data, moving beyond a categorical, dichotomous approach rooted in previous diagnostic models, such as DSM-IV-TR or ICD-10 (American Psychiatric Association, 2000; World Health Organization, 2007; Negu et al., 2016), and the distinction between aMCI and naMCI.

The exclusive implementation of neuropsychological assessment tools cannot provide information on the finer behavioral aspects of the early stages of MCI and, despite their widespread use and efficacy, they fail to predict an individual’s behavior in real life, and there is a need to improve their ecological validity, sensitivity, and specificity (Rizzo et al., 2004; Negu et al., 2016; Plancher and Piolino, 2017; Kim et al., 2019). The available objective methods for assessing MCI are frequently based on informant-reports or conducted in isolated and artificial situations, thus opening the possibility for evaluation biases. A resounding change might be fostered by a novel approach assembling in new ways existing technologies and data analysis methodologies that allow a refined assessment and the creation of a continuum for MCI following a dimensional approach. These technologies aim to integrate rather than replace existing neuropsychological evaluation or caregiver/informant reports in order to obtain a more complete and dynamic picture of the strengths and critical aspects of the elderly person as they evolve over time.

This perspective aims to propose the development of a new integrated, multimethod, dimensional approach for early detection of MCI on the basis of behavioral data that incorporates existing, consolidated technologies, such as gait kinematic analysis, Virtual Reality (VR), and Machine Learning (ML), in the conventional assessment of MCI. The outcome would be a finer, continuous, time-sensitive assessment of MCI, in line with a dimensional approach compliant with new DSM-5 guidelines (American Psychiatric Association, 2013). Moreover, it would draw on recent empirical evidence and scientific groundwork, helping the clinician to tailor the rehabilitation to the needs of the individual. This positive contribution would improve their quality of life, decreasing both health care assistance costs and hospitalization rates, thus opening up new possibilities for primary and secondary prevention. Moreover, it would facilitate the communication between practitioners and researchers, providing a solid foundation and fostering mutual exchange.

## A New Integrated Approach to MCI Assessment

We suggest that Virtual Reality would provide the most suitable context (i.e., answering the question *Where?*) for the assessment of key behavioral variables indicating MCI onset (i.e., *What?*), which can be analyzed in a systematic and accurate way in relation to neuropsychological and clinical data by means of Machine Learning (ML) (i.e., *How?*). We expand on all of these aspects in the following sections.

### “Where” Does the Assessment Take Place? Virtual Reality

Usually, the assessment of cognitive functions does not take place in daily-life contexts, potentially hindering an ecological evaluation of the individual’s impairment (Rizzo et al., 2004; de los Reyes-Guzmán et al., 2014). A promising integration of conventional practices could rely on novel dimensional assessment techniques, based on realistic immersive simulations of daily situations, e.g., Virtual Reality (VR) – a 3D computer-generated environment with some degree of immersion and interactivity, along with a sense of being really present in it (Riva and Mantovani, 2014; Riva et al., 2018, 2019a; Moreno et al., 2019). VR has developed into a key technology that is able to resemble even complex daily situations and interactions in a safe and controlled setting, due to the feeling of *immersion* (i.e., the number of senses stimulated within the environment, together with the closeness of the stimuli employed in simulations to reality) (Slater, 2009; Plancher and Piolino, 2017; Cipresso, 2018), the *sense of presence* within the environment (i.e., the feeling of being really “there” in the simulated environment, along with the ability to realize our intentions within it), and the possibility to *interact* with objects (Biocca, 1997; Heeter, 2000; Bailenson et al., 2006; Sundar et al., 2010; Negu et al., 2016; Plancher and Piolino, 2017; Cipresso, 2018; Kim et al., 2019). Depending on the degree of immersion of the system employed, VR allows a realistic experience through the use of multi-sensorial displays (i.e., visual, auditory) along with tracking devices that detect any movement of the individual and deliver the recorded data to the visualization system for a real-time update of the virtual environment (Chirico et al., 2016; Plancher and Piolino, 2017; Cipresso, 2018). The most immersive 3D VR environments can provide a high sense of presence also by isolating individuals, facilitating natural interactions and exchanges that resemble equivalent ones in daily life (Gold and Gold, 2012; Allain et al., 2014; Riva and Mantovani, 2014; Chirico et al., 2016; Riva et al., 2018).

The main features of VR allow the creation of ecological, safe, standardized settings and exert a strict experimental control over stimulus delivery and measurement (Rizzo et al., 2004; Gold and Gold, 2012; Allain et al., 2014; Negu et al., 2016; Plancher and Piolino, 2017). This, in turn, has supported its deployment for both clinical and non-clinical samples of elderly people and young adults (García-Betances et al., 2015; De Tommaso et al., 2016; Plancher and Piolino, 2017). Within medical and neuropsychological settings, VR has been extensively applied as an assessment and a rehabilitation tool for elderly people suffering from consequences of a traumatic brain injury (Aida et al., 2018; Alashram et al., 2019; Maggio et al., 2019), for post-stroke patients (Henderson et al., 2007; Saposnik and Levin, 2011; Laver et al., 2017), and for spatial memory and balance (Allain et al., 2014; Serino et al., 2017; Gerber et al., 2018; Soares et al., 2018), among other applications (see Plancher and Piolino, 2017; Moreno et al., 2019). Crucially, VR allows the therapy to be tailored in a controlled way, according to each disease starting from a continuous assessment of the individual’s behaviors. Only recently, VR has been employed to assess IADL in MCI patients while including kinematic measures that integrate a neuropsychological evaluation (Seo et al., 2017). As previously mentioned, an initial cognitive decline can be behaviorally manifested by a slowdown in the execution of IADL (Kim and Kim, 2009; Millán-Calenti et al., 2010; Gold and Gold, 2012), which implies a neurological and cognitive alteration that is partially reflected in indexes such as bodily movements or gait. Previous studies have examined these behavioral alterations of IADL in order to refine MCI assessment and have already delivered promising results (Schröter et al., 2003; Montero-Odasso et al., 2009; de los Reyes-Guzmán et al., 2014). Motion detectors, applied to the elderly person’s leg joints allow gait kinematics and their impairments to be tracked during the performance of IADL within a VR environment. This could consolidate preliminary findings of specific motor alterations that integrate neuropsychological and cognitive evaluation to identify MCI. In this perspective, the preliminary work of Seo et al. (2017) is the closest application of the technologies proposed to refine MCI assessment, although gait analysis was not included. The recording of kinematic measures from the performance of IADL within an immersive VR environment potentially adds more discriminative value in distinguishing MCI individuals from the healthy control group (Seo et al., 2017). Including an evaluation where the elderly person him/herself performs IADL might be essential for establishing more precise criteria (Díaz-Mardomingo et al., 2017; Seo et al., 2017). Several authors have tried to refine early MCI detection by combining two out of the three variables considered in this paper: either behavioral alteration (IADL, gait) within a VR environment (Lee et al., 2003; Seo et al., 2017; Kim et al., 2019; Eraslan Boz et al., 2019), gait kinematics extracted and analyzed by means of ML, which will be discussed further (Begg and Kamruzzaman, 2005; Pogorelc et al., 2012; Zhang and Wang, 2012; Eskofier et al., 2013; Akl et al., 2015; Costa et al., 2016; Mannini et al., 2016; Caldas et al., 2017; Farah et al., 2017; Ur Rehman et al., 2019), or ML techniques for predicting MCI evolution (Filipovych and Davatzikos, 2011; Williams and Weakley, 2013; Moradi et al., 2014, 2015; Bratić et al., 2018; Grassi et al., 2018, 2019; Graham et al., 2020). Thus, to our best knowledge, this is the first paper proposing an integration of VR, gait kinematics, and ML in order to refine early detection of MCI following a dimensional approach in line with the most recent diagnostic systems and possibly providing information on disease progression. However relevant, traditional neuropsychological assessment does not provide this extent of information and could serve as a starting point that should be integrated with further information in order to detect a subclinical condition otherwise undiagnosable following a categorical approach. Crucially, some anecdotal evidence and more systematic but scattered evidence from kinematic analysis of specific movements (i.e., *What?*) suggest the feasibility and the relevance of an approach based on assessment of behavioral variables for early detection of MCI. We present preliminary evidence in this regard in the following.

### “What” Variables Are Included in the Assessment? Gait Kinematics

Is it possible to give relevance to behavioral data reported by the caregivers, relying on the anecdotal description of the elderly person’s daily functioning and their IADL performance, in a scientific and rigorous manner?

A potential solution is to analyze the elderly individual’s movements (kinematics) while performing IADL. Kinematic analysis automatically records movements in a controlled setting and assesses the underlying cognitive impairment. Preliminary studies proved the feasibility of tracking the elderly person’s head, dominant hand, or gait during the performance of IADL to refine the assessment of MCI and other cognitive conditions (Schröter et al., 2003; de los Reyes-Guzmán et al., 2014; Akl et al., 2015; Seo et al., 2017). Among these indexes, gait kinematic analysis has progressively received more attention, despite the paucity of MCI-focused studies. The work of Martín-Gonzalo et al. (2019) thoroughly explains the contribution of considering gait alterations, beginning in early cognitive decline, to an improved understanding of neurocognitive disorders. In fact, gait kinematics are strongly related to neurophysiological alterations (Persad et al., 2008; Maquet et al., 2010; Martín-Gonzalo et al., 2019), brain volume changes in specific areas (Tian et al., 2017; Allali et al., 2019; Martín-Gonzalo et al., 2019), and subsequent cognitive decline, predicting future risks of impairment (Martín-Gonzalo et al., 2019). Kinematics assesses the sequential configuration of the leg joints required to maintain the body’s center of gravity above the stance base while a person is moving forward. Compared to healthy subjects, the gait of a person suffering from MCI shows decreased velocity, longer stride time, increased stride-to-stride variability (Hausdorff, 2007; Bahureksa et al., 2017; Byun et al., 2018; Martín-Gonzalo et al., 2019), and spatiotemporal complexity (Ihlen et al., 2016; Martín-Gonzalo et al., 2019).

A gait cycle is defined by ongoing changes in the sequential configurations of the joints allowed by muscle activation, which is controlled by neural mechanisms depending on the integrity of somatosensory, motor, and cognitive integration cerebral networks (Perry and Burnfield, 2010; Caldas et al., 2017; Costilla-Reyes et al., 2020). Successful locomotion is indeed a dual task requiring the ability to simultaneously perform a cognitive task that could interfere with gait performance, particularly in elderly people (Pedroli et al., 2018; Costilla-Reyes et al., 2020). A decrease in attentional and executive functioning is physiological in aging and could impact this simultaneous execution (Hsu et al., 2012; Montero-Odasso et al., 2012; Wang et al., 2015; Gwak et al., 2018; Pedroli et al., 2018). In order to maintain walking capacity, damage to cerebral networks involved in gait leads to an adaptation of the nervous system, generating new signals reflecting the damage. Brain signals to the muscles controlling joint movement may become discontinuous and uncoordinated: this generates noise that could be consequent to the failure of some neuronal networks and produces configurations that respond to intentional cognitive directives, such as changing gait pace, little or not at all (Martín-Gonzalo et al., 2019). Indeed, kinematic data provide additional, crucial information that increases the sensitivity and specificity of MCI assessment. Paper-and-pencil neuropsychological tests are not suitable for the detection of gait features and its alterations, which appear relevant for more precise identification of MCI individuals.

To date, gait analysis has been studied within a context with little ecological validity: the walking task is generally an end in itself and is not recorded while the subject is completing a complex activity. Even the extraction of gait kinematics from videos or home-based motion sensors could provide only partial, bi-dimensional information or could be less sensitive in detecting real-time movement adjustment (Akl et al., 2015; Prakash et al., 2015; Neverova, 2016). The use of VR enables continuous, tridimensional tracking of the ongoing events within a highly immersive, safe, and standardized environment, enhancing the methodological strength of the procedure as well.

**FIGURE 1:**
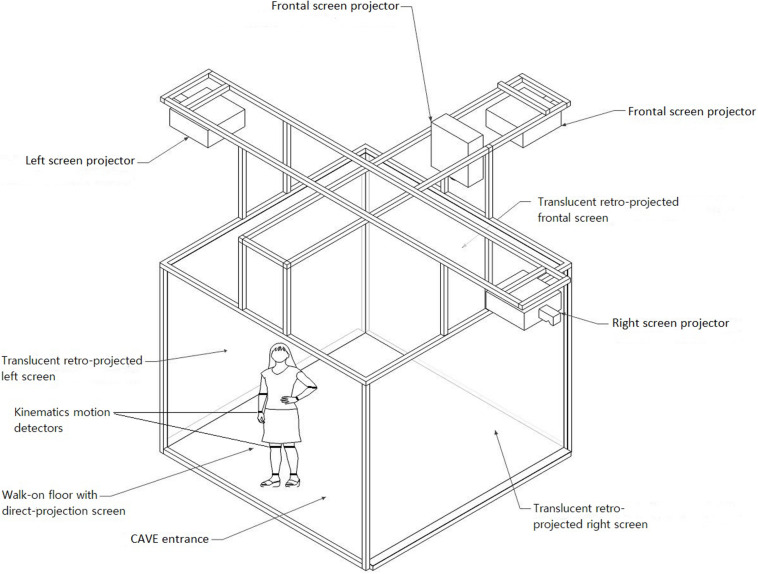
Subject’s kinematic measures (gait) detected within a VR environment (CAVE) while performing instrumental activities of daily life (IADL) in order to refine MCI assessment by following a dimensional approach.

This introduces the need for a highly ecological and immersive context that allows the elderly person’s kinematics while performing IADL to be observed and detected. A plausible solution comes from the implementation of Virtual Reality (VR), as shown in Figure 1 (Pedroli et al., 2018). The technological equipment illustrated in Figure 1 is a four-walled Cave Automated Virtual Environment (CAVE), available at Istituto Auxologico Italiano, which is routinely used for cognitive and motor rehabilitation of elderly people. This highly immersive technology is equipped with eight (4 × 2) Vicon Bonita 10 cameras (Opti-Tracking system, 1MP) and different Hi-res Hi-FOV head-tracked 3D HMDs and also with a wide range of physiological and motion measures for quantifying embodiment in VR and movements within the environment. A virtual representation of, e.g., a city or a supermarket can be projected on the four walls, and subjects can actively navigate and interact with the environment. This setting was used by Seo et al. (2017), which, as previously mentioned, is the most similar procedure to the one that we propose.

For the aims of this perspective, the most important feature of VR is its ability to detect both real-time behaviors (e.g., specific bodily movements such as those of the head and upper limbs and gait) and physiological indexes (e.g., skin conductance, heart rate). The great amount of data collected with VR and kinematics implies the need for a computational method of analysis that is able to extract meaning from a large amount of data. Thus, Machine Learning appears to be a viable solution, as shown by previous research employing this technique to discriminate between normal and pathological gait alteration and for diagnostic purposes as well (Pogorelc et al., 2012; Zhang and Wang, 2012; Eskofier et al., 2013; Costa et al., 2016; Mannini et al., 2016; Caldas et al., 2017; Farah et al., 2017; Ur Rehman et al., 2019).

### “How” to Analyze Them? Machine Learning

The massive amount of kinematic information extrapolated from motion detectors, complemented by neuropsychological and neuropsychiatric symptoms and signs, needs a similarly powerful technology in order to process it and convert it into an output intelligible for both clinicians and patients. The employment of kinematic measures and VR within a healthcare setting, i.e., a hospital, inevitably involves the use of a large amount of electronic health records (EHR) of patients’ evolution over time. Despite the challenges related to the use of EHRs, several prediction algorithms and models have been developed from their use (Häyrinen et al., 2008; Miotto et al., 2016, 2017; Goldstein et al., 2017; Graham et al., 2020). Among other advantages, EHR-based predictors consider various metrics of multiple individuals, observed at different time points: this makes use of a higher frequency of data recording, facilitating the prediction of possible near-term evolution; they also reflect real life more closely than cohort studies (Goldstein et al., 2017).

The most suitable technique capable of administering a volume of complex and extensive information may be Machine Learning (ML). This scientific discipline stems from Artificial Intelligence (AI), i.e., a computer science field performing tasks capable of emulating human performance, generally learning to understand complex data, an endeavor that requires human intelligence (Bawack, 2019; Wang, 2019; Graham et al., 2020). ML algorithms have progressively gained popularity for several reasons, including their ability to automatically learn the inherent structure of a dataset (Kononenko, 2001; Abu-Mostafa et al., 2012; Facal et al., 2019) without requiring *a priori* hypotheses about relationships between variables (Miotto et al., 2017; Vieira et al., 2017; Graham et al., 2019, 2020). Conversely, ML algorithms can discover and predict data trends and patterns by building on existing information and highlight unexpected relationships between variables (Vieira et al., 2017; Graham et al., 2019, 2020). This “learning by processing” approach generates increasingly accurate predictive models and, so far, has demonstrated enormous potential for supporting individual prognosis, risk estimation, and classification learning for diagnosis (Lehmann et al., 2007; Patel et al., 2015; Vieira et al., 2017; Dwyer et al., 2018; Facal et al., 2019). Inevitably, ML techniques work with high-dimensional data, which require a pre-processing step to remove redundant information, reduce data dimensionality, and improve learning accuracy and data comprehensibility (Khalid et al., 2014). This can be achieved by means of (i) feature selection (i.e., the selection of the best and most optimal features from a larger set of those useful for discriminating between classes to increase accuracy and generalizability); and (ii) feature extraction or dimensionality reduction (i.e., the transformation of original features to generate other, more significant features and reduce complexity) by means of Principal Component Analysis (PCA) or Independent Component Analysis (ICA), among other approaches (Khalid et al., 2014; Dwyer et al., 2018). The application of ML for healthcare purposes has been further developed into two main sub-classes, supervised (SL) and unsupervised (UL) techniques. SL jointly employs pre-labeled data, e.g., MCI versus healthy subjects, and additional features derived from clinical or neuroimaging sources to determine which feature predicts the pre-labeled data the most (Dwyer et al., 2018; Graham et al., 2020). SL operates with probabilistic and non-probabilistic classifiers (Naïve Bayes and Support Vector Machine, respectively), as well as with decision tree, linear, and logistic regression (Dhall and Kaur, 2020). UL techniques, instead, sets unlabeled and unstructured data, e.g., clinical notes, as a starting point to seek relationships or patterns and to learn general representations that enable the automatic extraction of information when building predictors (Miotto et al., 2017; Dwyer et al., 2018; Graham et al., 2020). The algorithms employed by UL include K-means clustering, PCA, and Artificial Neural Networks (ANN) (Dhall and Kaur, 2020).

However, at the time of data collection, it is unclear whether MCI subjects will progress toward other forms of dementia (e.g., AD) or convert back to normal cognition, and this evolution could become more evident over the years. This challenges data labeling: thus, researchers tackling MCI detection have employed semi-supervised learning (SSL) techniques capable of combining labeled and unlabeled data to improve the classification procedure (Zhu, 2008; Filipovych and Davatzikos, 2011; Moradi et al., 2015; Dwyer et al., 2018; Van Engelen and Hoos, 2020). A semi-supervised approach, therefore, allows cases to be managed by providing only partial data labels (Filipovych and Davatzikos, 2011). Several studies have employed MCI data as unlabeled data and have shown an improvement in the predictive performance of the model (Batmanghelich et al., 2011; Filipovych and Davatzikos, 2011; Ye et al., 2011; Moradi et al., 2015): this approach could be particularly feasible for the purpose of the integrated dimensional approach offered in the present perspective.

The ability to process raw data, the need for manual engineering of features, and the extensive expertise needed to perform the analyses represent the main limitations of conventional (shallow) ML techniques (Lecun et al., 2015; Vieira et al., 2017; Zhang et al., 2020). This has led to the dissemination of deep learning (DL) algorithms, including Deep Neural Networks (DNN), Convolutional Neural Networks (CNN), and Recurrent Neural Networks (RNN) (Dhall and Kaur, 2020). DL outperforms ML in many ways, showing best-in-class performance and increased complexity in the computed function and addressing problems in multiple domains such as language and speech (Zhang et al., 2020). Moreover, it eliminates the need for manual feature engineering, reducing possible human biases and removing the need for advanced expertise (Zhang et al., 2020). DL is capable of learning data representation in an unprocessed or raw form, and its high performance and expressive power in one specific domain can be transferred to other contexts, providing a flexible adaptation to problems (Bengio, 2009; Lecun et al., 2015; Miotto et al., 2017; Vieira et al., 2017; Chauhan et al., 2019; Esteva et al., 2019; Costilla-Reyes et al., 2020; Zhang et al., 2020). Despite all the advantages, it is crucial to consider that DL techniques require very large datasets to perform, which may be too hard to achieve, expensive, or time-consuming to obtain; thus, ML may be more feasible and efficient (Zhang et al., 2020).

To date, advanced statistical ML and pattern recognition techniques have proved their usefulness in outlining neurodegenerative patterns of mild symptoms manifesting during the early stages of diseases, and MCI is no exception (Davatzikos et al., 2008, 2010; Vemuri et al., 2009; Wee et al., 2014). ML has been repeatedly applied to diagnostic transitions from MCI to other forms of dementia, e.g., AD, employing different types of information: mostly neuroimaging data (e.g., MRI, PET scan, Diffusion Tensor Imaging) (Batmanghelich et al., 2011; Filipovych and Davatzikos, 2011; Ye et al., 2011; Zhang and Shen, 2011, 2012; O’Dwyer et al., 2012; Shaffer et al., 2013; Moradi et al., 2015; Bratić et al., 2018), cerebrospinal fluid biomarkers (Davatzikos et al., 2010; Fjell et al., 2010; Zhang and Shen, 2011; Shaffer et al., 2013; Bratić et al., 2018), demographic and cognitive data (Moradi et al., 2015; Bratić et al., 2018), and gait kinematics (Mannini et al., 2016; Farah et al., 2017; Gwak et al., 2018). Broad variations in studies’ results have been reported, as has the lack of a gold-standard ML algorithm to predict disease progression (Grassi et al., 2018, 2019; Chiu et al., 2019; Facal et al., 2019; Mallo et al., 2019). Specifically, Grassi and colleagues (Grassi et al., 2018, 2019) have recently developed clinically translatable ML algorithms to identify which subjects with pre-MCI and MCI will convert to AD (Grassi et al., 2018, 2019). ML likewise appears promising for precision medicine: given the patients’ extreme heterogeneity of symptoms, medication response, and prognosis, the implementation of ML to create computational models of disease development tackles patients’ diverseness (Fisher et al., 2019). Over the years, researchers have devised a number of disease progression models for both MCI and AD, relying on clinical and imaging data (Mueller et al., 2005; Ito et al., 2011; Rogers et al., 2012; Moradi et al., 2015; Miotto et al., 2017; Samper-Gonzalez et al., 2017; Fisher et al., 2019). Previous applications of ML to clinical data have proven useful in predicting a single outcome (e.g., the likelihood of conversion from MCI to AD) (Fisher et al., 2019). From a clinical point of view, however, it would be important to predict the disease progression and trajectory for everyone, which is difficult with current data-driven modeling approaches.

In their latest work, Graham et al. (2020) support the employment of AI and ML for ranking those variables crucial for MCI assessment and cognitive impairment. The authors show that clinical and psychometric assessments appear promising for identifying individuals at high risk for cognitive impairment (Lins et al., 2017; Senanayake et al., 2017; Moreira and Namen, 2018), which could be better identified by means of brain imaging and neuropsychological data as well (Fan et al., 2018; Iizuka et al., 2019). Even more importantly, Graham et al. (2020) report on several studies employing novel techniques to detect cognitive impairment in MCI subjects as well, such as home-installed motion sensors (Akl et al., 2015) and multi-modal wearable activity devices (Gwak et al., 2018), therefore including behavioral data in ML analysis (Graham et al., 2020). However useful, for providing real-world behavioral data in an ecological context, the employment of motion sensors alone has shown substantial heterogeneity (Graham et al., 2020); therefore, VR appears a promising integrative solution achieved by simulating a supervised and controlled real-life-like environment.

## Discussion

Although both elderly people and caregivers notice and report their concerns regarding behavioral, personality, and cognitive changes, MCI is a subclinical condition that remains undiagnosed by an official categorical system while progressively compromising the independent functioning of the elderly person. Although a possible regression to normal cognition is desirable, more often, MCI evolves toward other forms of dementia. A delayed diagnosis entails the worsening of the individual’s conditions, greatly reducing the extent of possible interventions and making primary and secondary prevention essential (Van Vliet et al., 2011; Jekel et al., 2015; Roehr et al., 2019). However, MCI assessment should necessarily move beyond a stringent categorical approach in favor of a dimensional one able to include finer discrimination among early stages of MCI, thus reflecting the complexity of this construct. So far, its assessment has followed a dichotomous view, relying on neuropsychological instruments to test MCI’s presence or absence. Despite their proven efficacy, a dimensional approach would integrate them by implementing existing technologies and data analysis methodologies, placing MCI on a continuum. With this in mind, this perspective aimed primarily to move forward, proposing a novel assessment that could enable a more accurate prevision of the trajectory of MCI decline, employing Virtual Reality (VR) for a continuous dimensional assessment of MCI behaviors in ecological and realistic tailored, safe, and controlled simulated contexts.

Since the individual’s altered behavior reflects impaired cognition (Martín-Gonzalo et al., 2019), this proposal would allow early detection of MCI, enabling timely rehabilitative interventions. Specifically, gait kinematics is a behavioral index whose analysis has proved sensitive to cerebral and cognitive alterations capable of discerning patients with cognitive decline from healthy individuals (Martín-Gonzalo et al., 2019). Nevertheless, few studies have specifically employed gait measurement as a possible marker to refine MCI assessment, and even then mainly in unfamiliar contexts, thus hindering ecological validity (Jekel et al., 2015; Seo et al., 2017). So far, MCI assessment has relied on neuropsychological measures rather than behavioral ones, despite the importance of the latter in revealing initial cognitive decline. When available, these behavioral data are generally based on informant-report questionnaires or reported as anecdotal information lacking scientific rigor (Van Vliet et al., 2011; de los Reyes-Guzmán et al., 2014; Kim et al., 2019). Behavioral data appear to provide a relevant contribution to MCI assessment: further research should deepen and consolidate the preliminary, promising evidence reported (Seo et al., 2017; Martín-Gonzalo et al., 2019).

**FIGURE 2:**
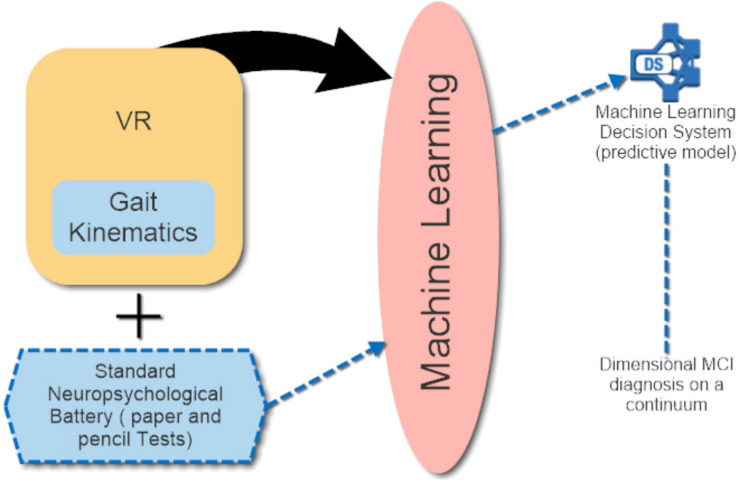
Schematic illustration of the innovative model proposed.

It appears evident that a mere conventional neuropsychological assessment, however relevant, cannot provide such a high degree of information, giving rise to the necessity of integrating paper-and-pencil instruments and anecdotal evidence with behavioral alterations evaluated within a highly ecological and standardized setting, such as VR. A plausible, practical implementation of the approach could be structured as follows. During a first brief clinical interview, the practitioner could collect anamnestic and quantitative information from (i) the elderly person, relying on neuropsychological/neuropsychiatric and cognitive measures as well; and (ii) the caregiver, which could fill in informant-report IADL measures. A second appointment would be dedicated to VR-based assessment: the elderly person could perform IADL (e.g., money withdrawal, grocery shopping) within the CAVE virtual environment, while kinematic information of their performance would be simultaneously collected. These data could be provided by kinematics motion detectors placed on the individual’s joints, as illustrated in Figure 1. The entire VR-kinematic assessment would last a maximum of 20 min to possibly avoid cybersickness, i.e., a form of motion sickness that includes nausea, headaches, and disorientation, among other symptoms (Laviola, 2000; Davis et al., 2014). Cybersickness is a common side effect of VR and could interfere with the completion of quantitative measures: thus, whenever it is necessary to complete paper-and-pencil assessment in the second appointment, this should be done before the VR procedure starts. VR would allow the clinician to closely observe the real life-like behavior of the individual and employ motion detectors, which extrapolate a large amount of data computed by means of Artificial Intelligence (AI) and, specifically, Machine Learning (ML). As mentioned in the ML section, recently developed, clinically translatable ML algorithms could help to identify MCI subjects who will convert to AD (Grassi et al., 2018, 2019). Thus, these algorithms could be tested and implemented in the assessment procedure illustrated in the previous section after collecting data from both the quantitative evaluation and the VR procedure within the CAVE. This could generate an accurate, predictive model proposing a gradient of behavioral and cognitive decline: a subclinical condition such as MCI could not be detected promptly by a categorical approach. A schematic illustration of this model is depicted in Figure 2.

The first and foremost added value of this approach lies in moving one step forward toward refined MCI early detection by integrating (i) behavioral (gait kinematics, IADL), neuropsychological/neuropsychiatric and cognitive information; (ii) a highly ecological and standardized setting, such as VR; and (iii) a powerful method capable of analyzing an extensive amount of data and predicting MCI progression over time. This is the first dimensional approach jointly considering all of the mentioned sources of information, whether previous studies considered only two out of three variables at the same time. The main focus is the relevance of building an innovative assessment procedure that is data-fusion-based and capable of identifying a subclinical condition that is otherwise undetected. Many ML and DL algorithms exist to analyze the extensive amount of data collected and, except for some that were recently tested (Grassi et al., 2018, 2019), there is no consensus regarding a gold-standard algorithm to predicting MCI diagnostic transition. Moreover, several open-source libraries for ML can provide information regarding the most feasible programming language (e.g., Python) and algorithms to use (Rathi, 2019).

We are aware that the integration of kinematic analysis, VR, and ML, could be very expensive and may not be available in a clinical setting, such as in a hospital. In addition, there may be risk of initial acceptance resistance by elderly individuals and healthcare providers due to the novelty of the equipment. However, the implementation of this approach would offer a crucial benefit by enabling the dimensional assessment of a subclinical condition otherwise undiagnosable, and the trained models, enriched by data of numerous patients, would easily overcome the initial expense. Moreover, a hospital would be the only setting where biological and neuroimaging data (e.g., MRI, PET scan, cerebrospinal fluid biomarkers) can be collected. Although the method proposed, so far, does not include them, these types of information could eventually be added to ML analyses, since they have been previously indicated as plausible contributors to MCI assessment (Fjell et al., 2010; Batmanghelich et al., 2011; Filipovych and Davatzikos, 2011; Ye et al., 2011; Zhang and Shen, 2011, 2012; O’Dwyer et al., 2012; Shaffer et al., 2013; Moradi et al., 2015; Bratić et al., 2018; Chiu et al., 2019). The employment of ML and DL methods usually requires a large sample size, which may not always be feasible in the healthcare setting. However, this limitation could be settled by developing multi-centric studies, providing an adequate sample size of patients and sharing data (Vieira et al., 2017). The dimensional approach also needs to be applied carefully in order to avoid hypervigilance for the slightest cognitive and behavioral age-related alteration, which might lead to excessive diagnosis and false-positives (Vanacore et al., 2017). Diagnosis communication must be carefully handled, given the potential harm of anxiety about a condition that may not progress (Chiu, 2005; Díaz-Mardomingo et al., 2017; Vanacore et al., 2017) prognostic possibilities can be discussed and planned accordingly. Strengthening or rehabilitative interventions could foster regression to normal cognition or decelerate the progression toward other clinical conditions.

In summary, while the majority of the literature has studied the application of several combinations of VR, gait kinematic analysis, and ML, this is the first paper to integrate all of these three methods and techniques in order to refine early detection of MCI and possibly predict its evolution over time. VR allows the collection of “Digital Biomarkers” – physiological/behavioral data - by means of digital technologies, used as an indicator of biologic processes or responses to therapeutic interventions (Coravos et al., 2019) directly connected to brain functioning. On the other side, AI, by applying ML techniques to the individual’s digital biomarkers, allows the creation of a predictive model – following a dimensional approach to MCI – able to identify specific behavioral cognitive patterns within an ecological and safe environment, for accurate early detection of MCI and its potential evolutionary trajectory (Riva et al., 2019b).

## Author Contributions

SC, AC, and PC developed the new model integrating gait kinematics, Virtual Reality, and Machine Learning. PC and GR supervised the sections of Virtual Reality and Machine Learning. EP supervised the section regarding MCI. SC wrote the manuscript under the final supervision of AC, EP, GR, and PC. All authors have approved the final version of the manuscript.

## Conflict of Interest

The authors declare that the research was conducted in the absence of any commercial or financial relationships that could be construed as a potential conflict of interest.
